# Supplementary material for: A Randomized Controlled Dietary Intervention Improved the Serum Lipid Signature towards a Less Atherogenic Profile in Patients with Rheumatoid Arthritis
Source: Metabolites. 2021 Sep 17;11(9):632. doi: 10.3390/metabo11090632 (PMC8472309; doi:10.3390/metabo11090632)
Supplement: Supplementary file 1 [file metabolites-11-00632-s001.zip › metabolites-1375887-supplementary.pdf]

Supplemental files:

Table S1. Effects of dietary intervention on lipid classes and scores associated with Cardiovascular risk.

Lipid class	Intervention Median (IQR)[a] (n=45)	Control Median (IQR)[a] (n=44)	Post period difference (n=43)	Pb Intervention vs control (n=46)	Pc Intervention vs control (n=28)
AC	−0.027 (−1.360, 0.965)	−0.026 (−1.172, 1.13)	−0.258	0.513	0.692
CE	3660 (−11500, 27500)	−7466 (−22360, 8767)	6560	0.125	0.268
Cer	−0.276 (−1.070, 0.459)	0.286 (−0.313, 1.009)[d]	−0.659	0.001	0.001
DAG	−0.57 (−8.03, 3.79)	−1.209 (−4.96, 4.878)	−2.06	0.282	0.949
Gb3	0.210 (−1.250, 1.620)	0.495 (−1.247, 1.981)	0.443	0.714	0.505
Glc/GalCer	0.197 (−0.288, 0.724)	−0.461(−0.837,0.583)	0.138	0.175	0.220
Lac Cer	0.324 (−0.763, 1.21)	−0.043(−0.649,0.689)	0.040	0.310	0.890
LPC	−23.8 (−86.8, 88.7)	7.221(−114.668,174.258)	−45.1	0.255	0.652
LPCO	−0.167 (−0.981, 0.928)	0.502(−1.1,1.642)	−0.642	0.530	0.845
LPCP	−0.047 (−0.223, 0.195)	0.162(−0.214,0.444)	−0.119	0.024	0.087
LPE	−0.76 (−3.19, 2.33)	−0.778(−3.439,2.806)	−0.374	0.505	0.895
LPEP	0.045 (−0.340, 0.279)	0.059(−0.39,0.43)	−0.112	0.116	0.098
LPI	0.020 (−0.309, 0.325)	0.058(−0.261,0.406)	0.007	0.679	0.963
PC	−49.4 (−151, 77.1)	16.0(−68.2,135.6)	−6.69	0.037	0.037
PCO	−5.68 (−13.0, 5.96)[d]	3.40(−7.48,14.12)	−6.86	0.005	0.003
PCP	−4.06 (−9.47, 4.11)	4.62(−6.97,12.74)	−6.24	0.022	0.005
PE	−1.99 (−7.58, 3.10)	1.63(−3.84,4.90)	−0.997	0.104	0.133
PEO	−0.006 (−0.312, 0.288)	−0.01(−0.54,0.466)	−0.183	0.661	0.320
PEP	−0.028 (−0.459, 0.379)	−0.127(−0.46,0.539)	−0.119	0.789	0.340
PG	−0.004 (−0.033, 0.016)	0.011(−0.012,0.033)	−0.006	0.029	0.027
PI	−0.06 (−2.72, 1.90)	1.692(−1.798,4.657)	−0.697	0.155	0.169
SM	−1 (−773, 549)	98.614(−520.506,616.378)	190.	0.910	0.943
TAG	−3.57 (−20.3, 8.37)	5.769(−9.207,15.039)	−8.63	0.014	0.145
Long TAG score	0.30 (−1.00, 1.36)	−0.645(−1.867,0.488)[d]	0.636	0.017	0.025
PC score	17.2 (−32.4, 54.5)	−3.073(−37.673,23.815)	26.0	0.008	0.047
CE score	6150 (−7020, 15800)	−5245 (−12008,5810)[d]	4310	0.009	0.077
Short TAG score	−2.29 (−6.03, 2.43)[d]	1.042(−1.841,6.228)[d]	−3.21	0.004	0.023

AC, acetylcarnitine; CE, Cholesteryl ester; Cer, Ceramide; DAG, Diacylglycerol; Gb3, glycosphingolipid globotriaosylceramide; Glc/GalCer, glucosyl/galactosyl ceramide; Lac Cer, Lactosylceramide; LPC, Lysophosphatidylcholine; LPC(O), Lysoalkylphosphatidylcholine; LPC(P), Lysoalkenylphosphatidylcholine; LPE, lysophosphatidylethanolamine; LPE(P), Lysoalkenylphosphatidylethanolamine; LPI, lysophosphatidylinositol; PC, Phosphatidylcholine; PC(O), Alkylphosphatidylcholine; PC(P), Alkenylphosphatidylcholine; PE, phosphatidylethanolamine; PE(O), Alkylphosphatidylethanolamine; PE(P), Alkenylphosphatidylethanolamine; PG, Phosphatidylglycerol; PI, Phosphatidylinositol; SM, Sphingomyelin; TAG, triacylglycerol; Long TAG score (TAG>54 carbons and >=5 double bonds; PC score (the sum of PC, LPC, PCP with >=5 of double bonds); CE score (CE>4 double bonds); short TAG score (TAG <50 carbons and <=4 double bonds). a) µmol/l, Interquartile Range (first quartile, third quartile) b) Mixed model comparing the diet periods c) Mixed model comparing the diet periods -sensitivity analysis d) significant within period by wilcoxon signed rank test p<0.05

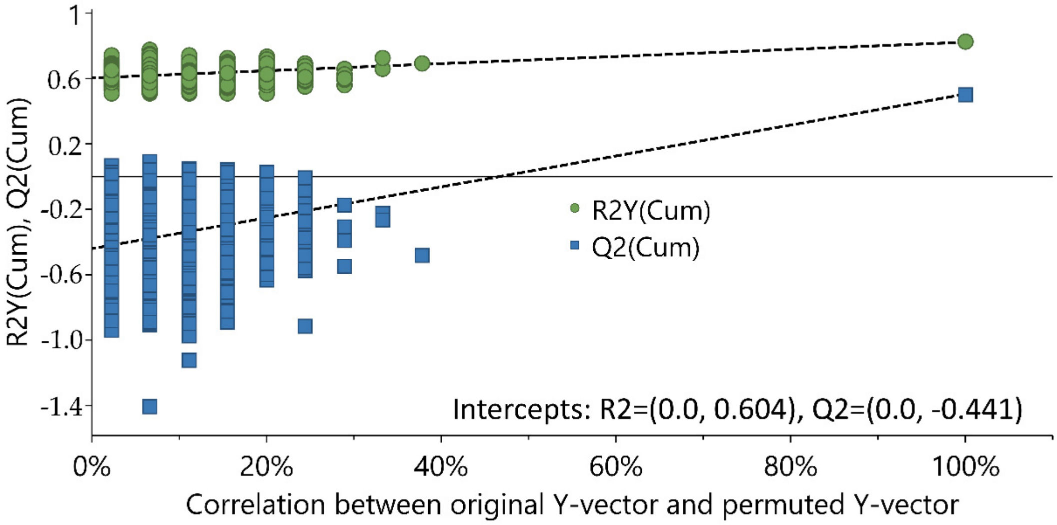

Figure S1. Permutation test OPLS-DA

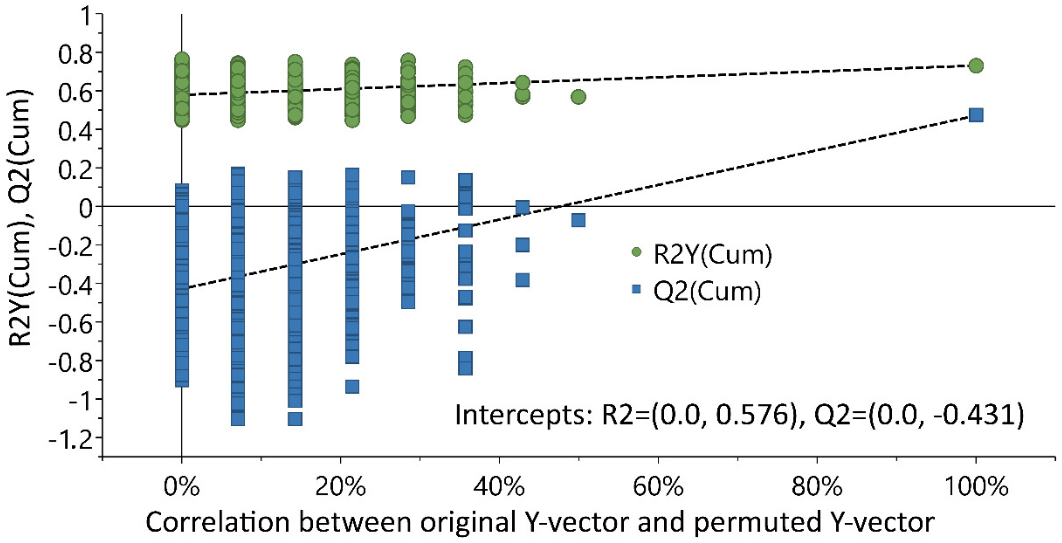

Figure S2. Permutation test OPLS-DA-S

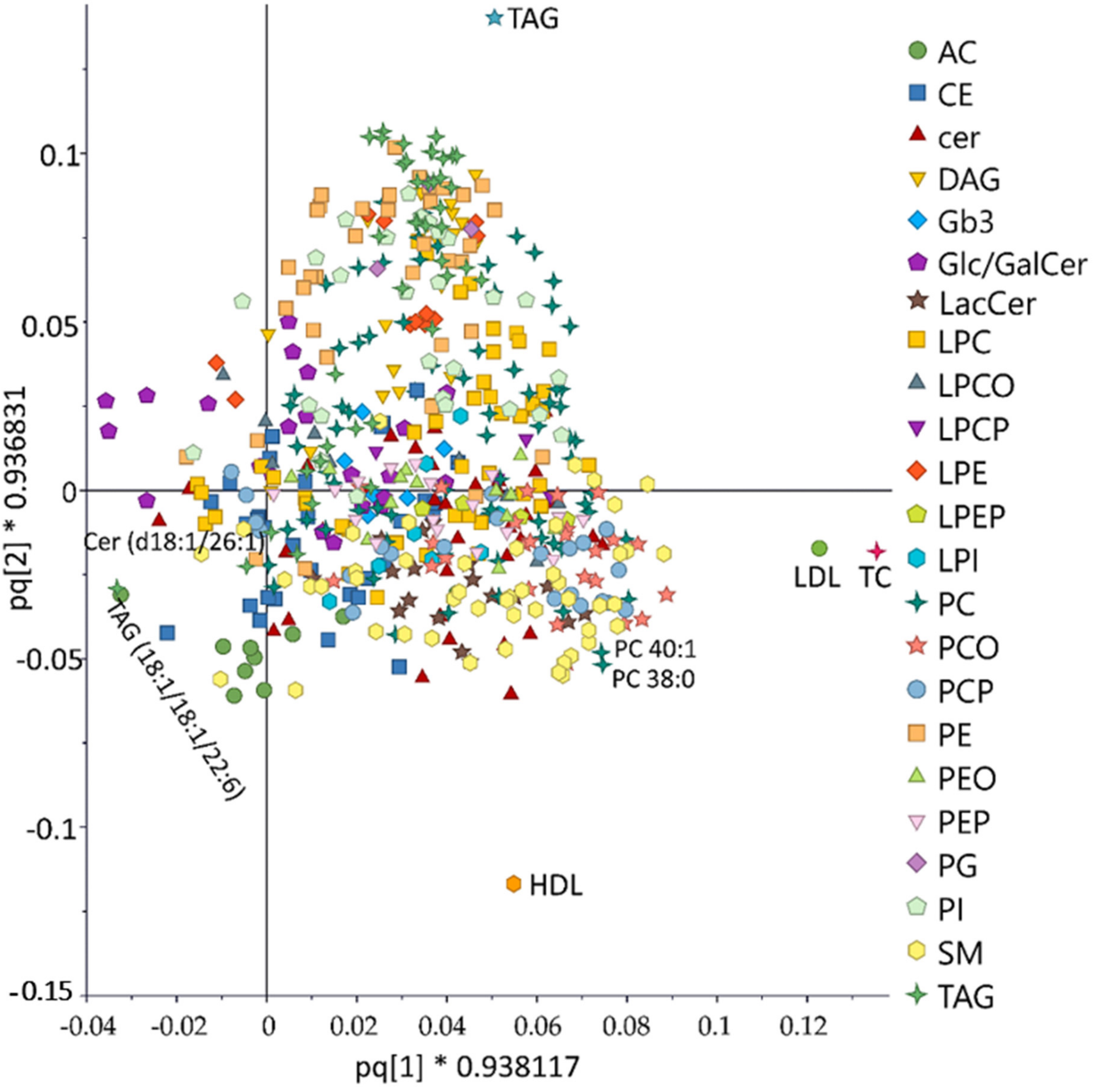

Figure S3. Loading scores for OPLS with clinical markers. TAG=triacylglycerides, TC= total cholesterol, HDL= high density lipoprotein, LDL=low density lipoprotein. Lipids that not cluster with their lipid class are named in the picture.

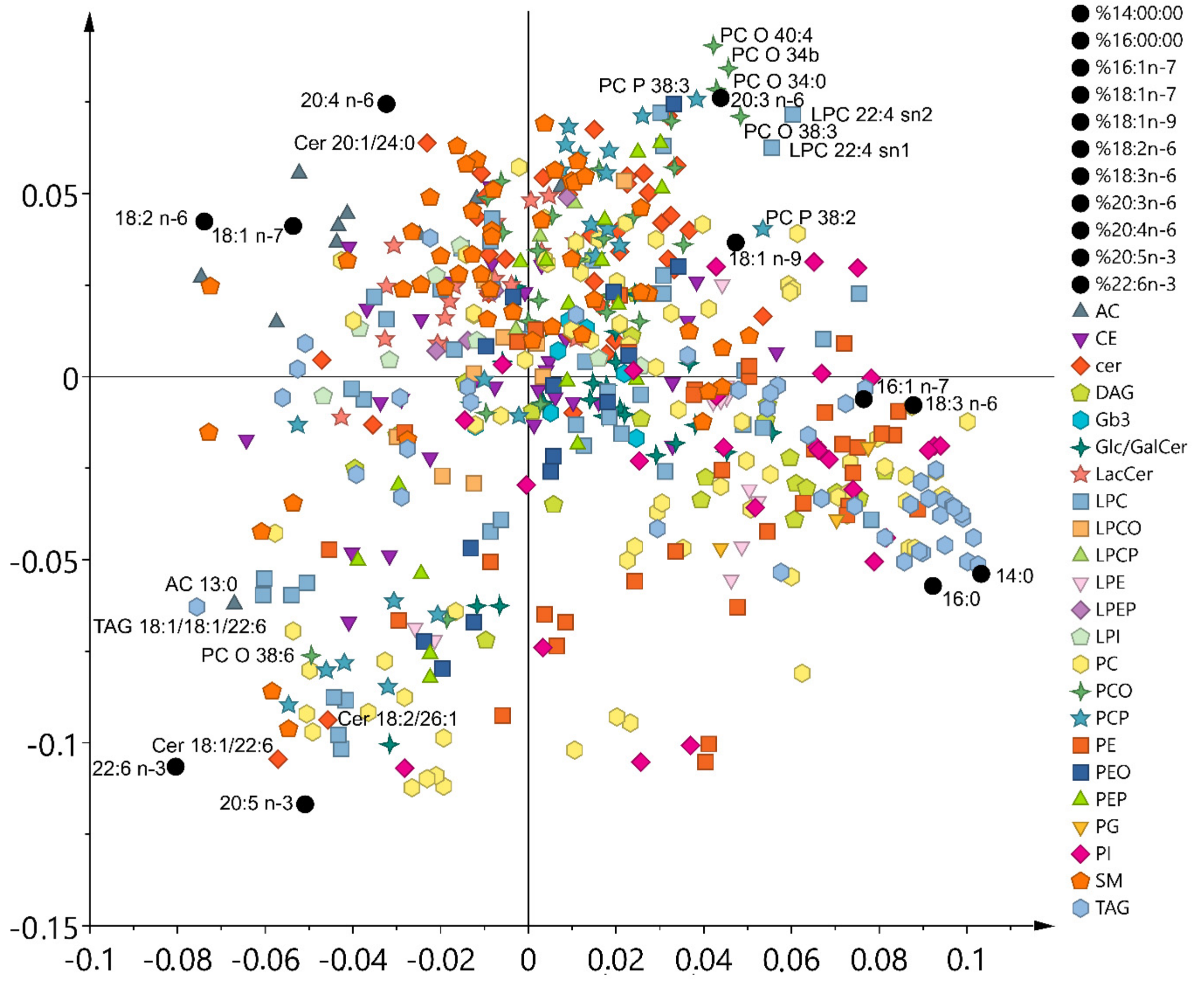

Figure S4. Loading scores for OPLS including fatty acids that were significant associated to the model. Fatty acids and lipids that not cluster with their lipid class are named in the picture.
